# Chronic Aortocaval Fistula Presenting as Right Heart Failure: A Case Report and Review of Literature

**DOI:** 10.7759/cureus.13528

**Published:** 2021-02-24

**Authors:** Sundeep Kumar, Akhil Mogalapalli, Mark R Milunski

**Affiliations:** 1 Cardiovascular Disease, Saint Louis University Hospital, St. Louis, USA; 2 Internal Medicine, University of Central Florida College of Medicine, Orlando, USA; 3 Internal Medicine, University Hospitals Cleveland Medical Center/Case Western Reserve University, Cleveland, USA; 4 Cardiology, Orlando Veterans Affairs Medical Center, Orlando, USA

**Keywords:** heart failure, right heart failure, aortic aneurysm surgery

## Abstract

Iatrogenic aortocaval fistula (ACF) is an infrequent cause of heart failure. A 65-year-old man presented to the cardiology clinic eight months after an open abdominal aortic aneurysm (AAA) repair. He developed predominantly right-sided cardiac failure after surgery, with minimal response to guideline-directed medical therapy. A transthoracic echocardiogram revealed decreased right-sided systolic function. A computed tomography angiographic scan of the abdomen revealed a large ACF at the distal end of the AAA repair. The patient was referred for closure surgery. ACF should be considered in a patient with unexplained right heart failure, especially in the setting of a known AAA or recent AAA repair.

## Introduction

We report an unusual case of refractory heart failure with predominant right-sided symptoms in a patient with previously documented moderately reduced left ventricular ejection fraction secondary to an iatrogenic aortocaval fistula (ACF). Aortocaval fistula is a rare complication that occurs when the aortic wall erodes into the wall of the inferior vena cava, resulting in a fistula. Cases with isolated heart failure symptoms without aneurysmal signs or rupture are rarely reported in the literature. 

## Case presentation

The patient is a 65-year-old gentleman who underwent elective open repair of a 6.5 cm abdominal aortic aneurysm (AAA) with a Dacron® graft eight months prior to his presentation. His postoperative course was uncomplicated, but soon after hospital discharge, the patient developed progressive lower extremity edema, generalized anasarca, and significant ascites requiring frequent high-volume paracentesis. He also had new persistent atrial fibrillation postoperatively, which was felt to be contributing to his heart failure (HF). However, cardioversion to sinus rhythm did not result in improvement of HF symptoms. He insisted that his symptoms developed soon after his AAA repair.

His past medical history is significant for essential hypertension, coronary artery bypass grafting and bioprosthetic aortic valve replacement, ischemic cardiomyopathy with a left ventricular ejection fraction (LVEF) of 35-40%, dual-chamber automated implantable cardioverter-defibrillator (AICD), and chronic obstructive pulmonary disease. Vital signs revealed a blood pressure of 120/50 mmHg. Physical exam revealed an elevated jugular venous pulse at 10 cm. Other findings included bilateral basilar crackles in the lungs, an S3 gallop, abdominal ascites, and significant bilateral lower extremity pitting edema. There was no abdominal bruit or pulsatile mass on abdominal examination.

**Figure 1:**
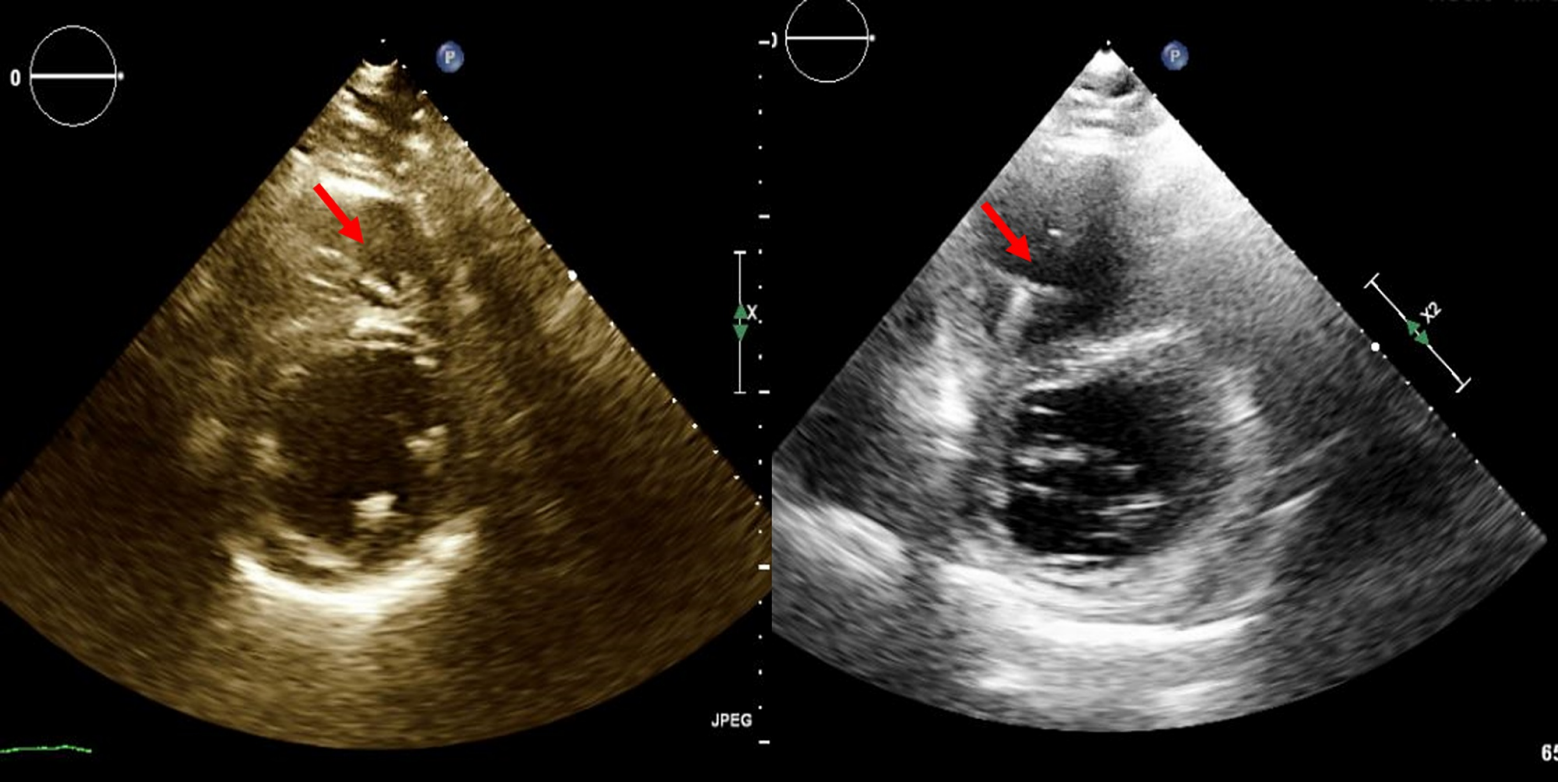
Transthoracic echocardiogram before (left) and after AAA repair (right), showing right ventricular dilation and flattening of the interventricular septum. AAA: abdominal aortic aneurysm

Laboratory studies showed a steady decline in renal function from normal baseline function, with doubling of serum creatinine over a span of six months. Brain natriuretic peptide (BNP) level was increased to 1800 pg/ml from a normal pre-AA levels (normal <100 pg/ml). He remained in New York Heart Association functional Class II-III despite compliance with guideline-directed medical therapy for HF. Because of his refractory HF symptoms, repeat echocardiography was performed and demonstrated severe right ventricular dilation with flattening of the interventricular septum consistent with right-sided pressure/volume overload, along with severely reduced right ventricular function. LVEF was unchanged from the pre-AAA repair range of 35-40%. Parasternal short-axis view of the right ventricle from the transthoracic echocardiograms before and after AAA repair are shown in Figure 1.

**Figure 2:**
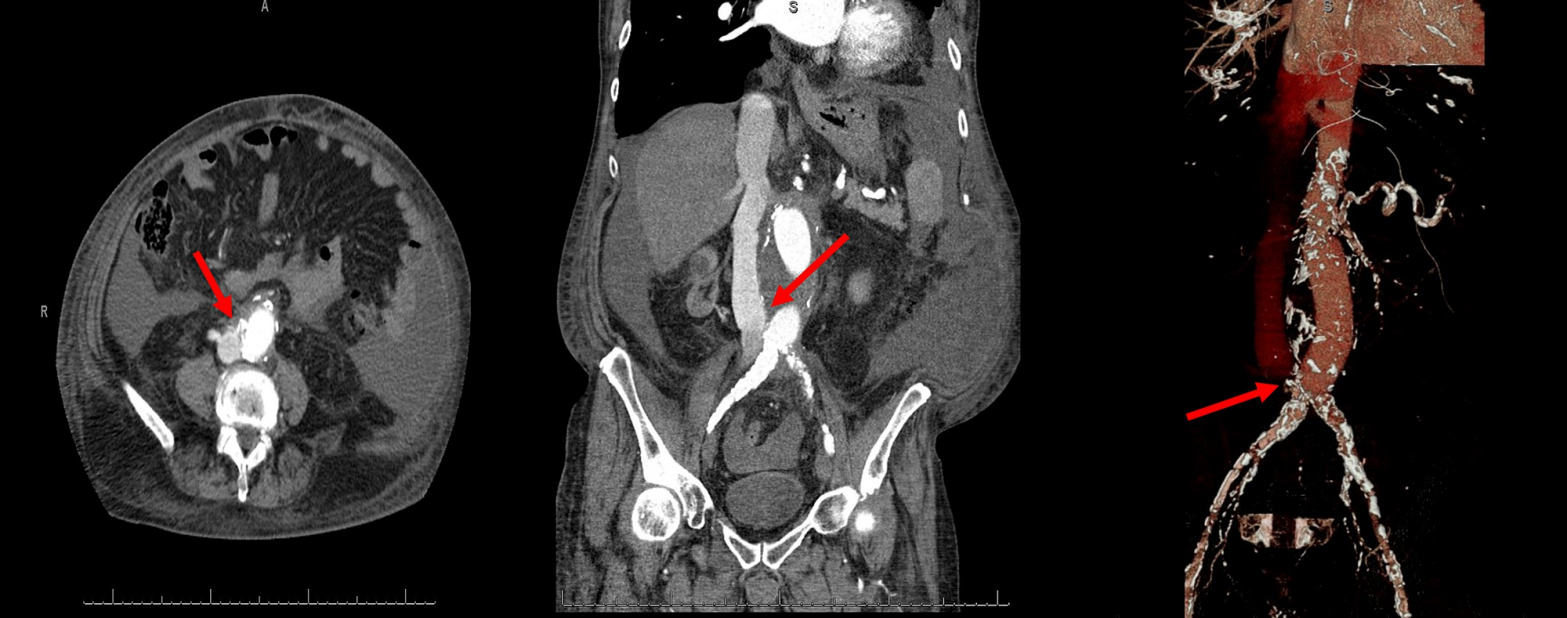
Computed tomographic angiography of the abdomen shows fistulous connection between AAA and inferior vena cava (red arrows) AAA: abdominal aortic aneurysm

In the course of being treated for an incarcerated abdominal hernia, he underwent an abdominal computerized tomography angiogram (CTA) that documented the presence of a large ACF at the distal end of the prosthetic aortic graft (Figure 2). It was after this procedure that he presented to our clinic. A vascular surgical consultation was placed and he is currently undergoing evaluation for the closure of the ACF.

## Discussion

Arteriovenous fistula, including aortocaval fistula, is one of the common causes of refractory heart failure [1]. They can be congenital, acquired, and iatrogenic. The earliest diagnosis of ACF dated back to the 18th century and was associated with nearly 100% mortality [2]. Almost all cases have been reported in men (~97%) [3]. ACF remained a fatal disease until 1955 when Dr. Denton Cooley performed the first successful repair [4]. Isolated cases have been described with fistulous connection occurring between the ascending or descending aorta and superior vena cava. However, the majority of cases involve the intrabdominal portion of the aorta and inferior vena cava. ACF is present in 5-6% of cases of ruptured AAA, and with less than 1% of non-ruptured aneurysms. The average diameter of AAA in cases with aortocaval fistula is 11 cm [3]. A proposed mechanism for ACF development implicates severe periaortic inflammation leading to adherence of an aneurysm to the inferior vena cava (IVC) with a resultant fistulous connection.

ACF can present with a wide array of signs and symptoms, including features of high output and right-sided heart failure [5]. Distinctive presenting symptoms include abdominal bruit, oliguria, hematuria, renal failure, back pain, wide pulse pressure, and regional venous hypertension, among others. Some case series have described hematuria as a common finding [6]. Acute renal failure as a presentation of ACF has been described [7]. Abdominal bruit is present in two-thirds of cases [3]. Only one-third of patients with ACF have features of heart failure [8-9]. Other signs of ACF such as bruits and pulsatile mass may not be present in nearly half of patients, thus potentially delaying the diagnosis [6,10]. 

Signs and symptoms of ACF depend on shunt flow, which in turn depends upon the size and chronicity of the fistula and the pressure gradient across the shunt. The overall impact of ACF depends on the patient’s overall functional status, including previous cardiovascular status, the proximity of the fistula to the heart, fistula dimension, flow rate through the fistula, and the timing of corrective surgery [11,12]. 

Diagnosis of ACF can be accomplished by color doppler ultrasound, computerized tomography angiography, magnetic resonance angiography, and aortography, which remains the gold standard [13-16]. Existing ACF is not recognized in nearly half of the patients undergoing AAA repair [17]. Acute cases of ACF require urgent surgical intervention. For patients with chronic ACF, the best treatment option may not be as clear. The role of medical therapy as the sole treatment is futile in the long term but may be supportive in patients awaiting corrective surgery. Patient-tailored, multidisciplinary care involving vascular surgery, interventional radiology, and cardiology should be undertaken for the best possible outcomes.

Increasing incidence of ACF has been recently reported, likely due to increased longevity, increased number of abdominal vascular procedures, and enhanced capability of diagnostic modalities. Patients with high output failure may get mislabeled as heart failure with preserved ejection faction because of higher estimated filling pressures (higher E/e’ ratio) seen with noninvasive testing [1]. It is imperative to segregate these individuals from patients at risk for high output failure as causative etiologies and treatment are different. Patients with high cardiac indices (>3.5 l/min/m2) and heart failure symptoms should include high cardiac output failure in the differential diagnosis [1]. Isolated or predominant right-sided symptoms should raise the suspicion for ACF and alternate etiologies even in presence of previous left heart failure. Chronic and potentially fatal sequelae of chronic ACF can be avoided by timely diagnosis and repair as the hemodynamic derangements usually improve after corrective surgery. ACF should be considered in a patient with unexplained right heart failure especially in the setting of a known AAA or recent AAA repair.

## Conclusions

We report a patient with known heart failure who presented with refractory predominantly right-sided heart failure symptoms post-AAA repair secondary to the aortocaval fistula. The patient was referred for endovascular repair of the aortocaval fistula. Aortocaval fistula should be considered in the differential diagnosis of isolated or predominant right-sided heart failure. Patients may not exhibit typical signs and symptoms of fistula and a high index of suspicion is required for timely diagnosis and management for ideal patient outcomes. 
